# High-affinity IgM^+^ memory B cells are defective in differentiation into IgM antibody-secreting cells by re-stimulation with a T cell-dependent antigen

**DOI:** 10.1038/s41598-018-32926-w

**Published:** 2018-09-28

**Authors:** Yasuyuki Tashiro, Akikazu Murakami, Yasushi Hara, Takeyuki Shimizu, Masato Kubo, Ryo Goitsuka, Hidehiro Kishimoto, Takachika Azuma

**Affiliations:** 1 Division of Development and Aging, Research Institute for Biomedical Sciences, Tokyo University of Science, Noda, Chiba, Japan; 2 Division of Biosignaling, Research Institute for Biomedical Sciences, Tokyo University of Science, Noda, Chiba, Japan; 3 Department of Parasitology & Immunopathoetiology, Graduate School of Medicine, University of the Ryukyus, Okinawa, Japan; 4 Shared equipment room, Research Institute for Biomedical Sciences, Tokyo University of Science, Noda, Chiba, Japan; 5 Department of Immunology, Kochi Medical School, Kochi University, Kochi, Japan; 6 Division of Molecular Pathology, Research Institute for Biomedical Sciences, Tokyo University of Science, Noda, Chiba, Japan; 7 Laboratory for Cytokine Regulation, Research Center for Integrative Medical Science (IMS), RIKEN Yokohama Institute, Yokohama, Kanagawa Japan; 8 Antibody Technology Research Center, Co. Ltd., Noda, Chiba Japan

## Abstract

IgM antibodies (Abs) are thought to play a major role in humoral immunity but only at the early stage of the primary immune response. However, two subsets of IgM^+^ memory B cells (MBCs), one with high affinity gained by means of multiple somatic hypermutation (SHM) and the other with low affinity and no SHMs, are generated through the germinal center (GC)-dependent and GC-independent (non-GC) pathway, respectively, after immunization with (4-hydroxy-3-nitrophenyl)acetyl (NP)-chicken γ-globulin. Surprisingly, an analysis of antibody-secreting cells reveals that a large amount of anti-NP IgM Ab with few SHMs is secreted during the recall response, indicating that only non-GC MBCs have terminal differentiation potential. Since secondary IgM Abs are capable of binding to dinitrophenyl ligands, they likely provide broad cross-reactivity in defense against microbial infection.

## Introduction

The (4-Hydroxy-3-nitrophenyl)acetyl (NP)-hapten system of the C57BL/6 mouse is advantageous for studying the structural bases of Ab affinities since anti-NP Abs are largely encoded by the *V186*.*2* gene, as well as by minor genes such as *V23*, *J558*.*55*, and *V124*, referred to as V_analogs_^1^. However, these immunoglobulin (Ig) heavy chains possess different CDR3 structures, particularly around the amino acid residue at position 95 that corresponds to the V_H_-D_H_ junction, where Tyr95 and Gly95 pair with His100 as well as X95 (where X indicates amino acids other than Tyr95 and Gly95)^2,3^. The structure at position 95 has been shown to contribute to variations in initial affinities^1,4^ and to raising the maximum affinity attainable by somatic hypermutation (SHM), namely, the ceiling affinity^5^.

By leveraging the NP system with a strong understanding of Ab affinity maturation, hapten-chromophore proteins with different numbers of hapten molecules, such as NP_hi_- or NP_med_-allophycocyanin (APC), have often been used to estimate the relative affinities of BCRs^6,7^. Using these NP-APCs, Hara *et al*. showed that there are two subsets of IgM^+^ memory B cells (MBCs) in the spleens of mice immunized with NP-chicken γ-globulin (NP-CGG): one expressing BCRs with increased affinities due to SHM, similar to those of IgG1^+^ MBCs acquired through a germinal center (GC) reaction (referred to as MBC^GC^), and the other expressing BCRs lacking SHMs that are supposedly generated through the GC-independent (non-GC) pathway (referred to as MBC^non-GC^)^7^.

Classically, MBCs are defined as B cells that have undergone antigen-driven proliferation and become resting cells. Upon re-exposure to primed antigens, MBCs are considered to proliferate and differentiate into Ab-secreting cells (ASCs)^8,9^. The expectation is that plasma cells secreting high-affinity IgM Abs are generated by secondary immunization; however, Murakami *et al*. showed that IgM Abs secreted from hybridomas prepared by cell fusion technology are characterized by consistently low affinity and few SHMs^10^. The results are in agreement with previous findings that IgM^+^ MBCs mainly comprise SHM^−^ cells^11–13^. To explain the discrepancy in maturation in terms of affinities between BCRs on MBCs^GC^ and secreted Abs from ASCs, an analysis of MBCs and ASCs at the same time points of immunization with NP-CGG was considered necessary.

Here, we report that IgM^+^ SHM^+^ MBCs^GC^ are unable to differentiate into IgM ASCs. On the other hand, IgM^+^ SHM^−^ MBCs^non-GC^ are capable of differentiating into plasma cells that secrete IgM Abs as part of the recall response. We also show that primary and secondary IgM Abs have different CDR3 structures that bring about different cross-reactivities to antigens; the secondary IgM Abs generally have very low initial affinities and can cross-react with the dinitrophenyl (DNP) hapten. Since neither primary IgM Abs nor affinity-matured IgG1 Abs bind to DNP, the broad cross-reactivity observed is considered to be characteristic of secondary IgM Abs, and their immunological role is discussed.

## Results

We analyzed the number of NP-specific B-lineage cells as well as Ab affinity with time after immunization with NP_40_-CGG by dividing the time course into three phases: Phases I, II, and III^14^. Phase I involves naïve B cell activation with antigens and the production of primary Abs by day 7. Phase II corresponds to the subsequent period up to the time of secondary immunization. GC structures are known to reside in follicles at this phase^15^. Phase III starts with secondary immunization, which induces MBC activation followed by MBC differentiation into ASCs that secrete secondary Abs.

### Kinetic analysis of activated B cell differentiation into MBCs and plasmablasts in Phases I and II

**Figure 1:**
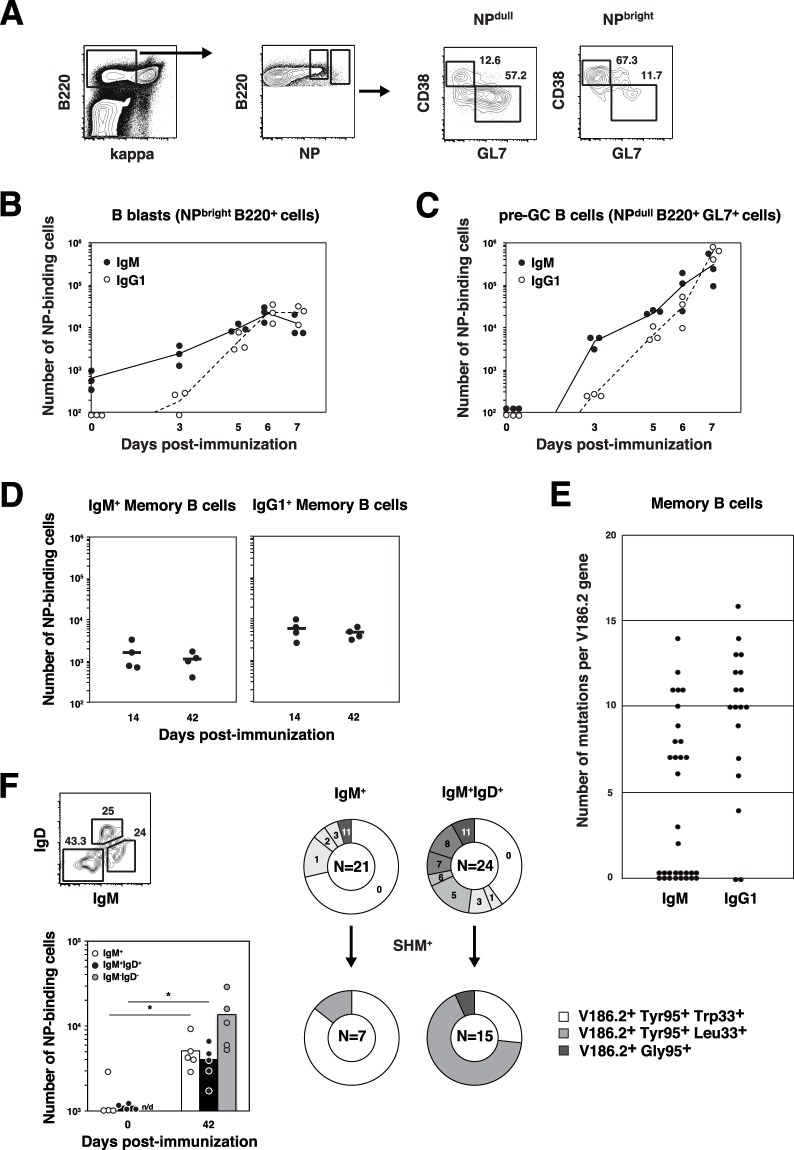
Analysis of the cell number and SHM frequency of B cells after immunization with NP_40_-CGG in Phase I and Phase II. (**A**) Flow cytometry of NP-specific (NP_hi_^+^ Igκ^−^) B cells in the spleen 7 days postimmunization with NP_40_-CGG/alum. The B cells were separated into NP^bright^ and NP^dull^ fractions, followed by the gating of GL7^+^ CD38^−^ cells or GL7^−^ CD38^+^ cells. The numbers in outlined areas indicate the percentages of GL7^+^ CD38^−^ cells (middle right) and GL7^−^ CD38^+^ cells (top left). (**B**) Changes in the number of NP-specific B cells (NP_hi_-APC-binding B220^+^ cells) with time postimmunization. (**C**) Changes in the number of pre-GC B cells (NP_hi_-APC-binding B220^+^ GL7^+^ cells) with time postimmunization. (**D**) Comparison of the numbers of GL7^−^ B cells on days 14 and 42. Right panel, IgM^+^ B cells; Left panel, IgG1^+^ B cells. (**E**) Comparison of the number of SHMs per *V186*.*2* gene between IgM^+^ and IgG1^+^ MBCs on day 42. Data were pooled from four independent experiments with one mouse per experiment in (**E**). (**F**) Flow cytometry of antigen-specific (NP_hi_^+^ Igκ^−^) B cells in the spleen on day 42 postimmunization with NP_40_-CGG/alum. The B cells were separated into three fractions based on the expression of IgM and IgD BCRs. Comparison of the numbers of IgM^+^ IgD^−^ cells (white bars) and IgM^+^ IgD^+^ cells (black bars) on days 14 and 42. Fractional ratios of B cells with different numbers of SHMs are compared between IgM^+^ IgD^−^ and IgM^+^ IgD^+^ cells. Fractional ratios of SHM^+^ V186.2^+^ B cells are divided into three based on the amino acid residues at positions 33 and 95. The results are presented as pie graphs pooled from five independent experiments with one mouse per experiment. The number of V_H_ sequences analyzed is indicated in the center. *p < 0.05. Data are from three independent experiments with one mouse per time point indicated in the figures for each experiment (**A**–**C**), from four independent experiments with one mouse per time point indicated in the figure for each experiment (**D**) or from 4–5 independent experiments with one mouse per time point indicated in the figures for each experiment (**F**).

First, we analyzed the cell fate of IgM^+^ B cells in Phase I. NP-specific IgM^+^ B cells were detected as NP^bright^ B220^+^ cells with NP_hi_-APC (Fig. 1A), although they were below the detection limit in unimmunized- and CGG-immunized mice (data not shown). This indicated that NP-specific naïve B cells were stimulated with NP_40_-CGG/alum and proliferated rapidly soon after immunization (Fig. 1B), followed by differentiation into either GL7^−^ B cells or GL7^+^ B cells by day 7 (Fig. 1B,C). Since IgG1^+^ B cells were also detected on day 3, class-switch recombination (CSR) was thought to occur at the early stage of immunization^7,16^. GL7^+^ B cells were also detected on day 3 as NP^dull^ B220^+^ cells (Fig. 1C), which would be pre-GC B cells destined to enter into GC structures that were not observed in follicles at this time point^15,17^. Since SHM was shown to be induced in GL7^+^ B cells on day 7 but not on day 5^7^ (Tashiro *et al*., unpublished observation), we distinguished pre-GC B cells from GC B cells on the basis of SHM expression. In other words, we used the SHM frequency as an index of B cells that had resided for a time in GCs.

We identified MBCs as NP_hi_-APC-binding GL7^−^ B220^+^ cells, and their number remained almost constant throughout Phase II (14 to 42 days postimmunization) (Fig. 1D). V_H_ sequence analysis of MBCs on day 42, when the GC reaction was almost complete, revealed two subsets: MBCs that had no SHMs (SHM^−^) and MBCs with multiple SHMs (SHM^+^) (Fig. 1E). Since pre-GC B cells had converted to GC B cells and since SHM was induced only in GC B cells, SHM^−^ GL7^−^ B cells were considered to be MBCs^non-GC^. Although both of these subsets evidently resided among IgM^+^ MBCs, there were only a few SHM^−^ cells among IgG1^+^ MBCs, suggesting that the IgG1^+^ fraction largely comprised MBCs^GC^ (Fig. 1E).

Next, we sorted the NP_hi_-APC-binding IgM^+^ MBCs into IgM^+^ IgD^+^ and IgM^+^ IgD^−^ cells according to a report by Dogan *et al*.^11^. These cells had increased in number on day 42 (Fig. 1F). The nucleotide sequences of the *V186*.*2* genes showed that the IgM^+^ IgD^+^ fraction contained more SHM^+^ cells than the IgM^+^ IgD^−^ fraction. In addition, since SHM^+^ cells in the IgM^+^ IgD^+^ fraction contained those with a Trp33Leu mutation (Leu33^+^), which was shown to increase affinity^18,19^, and since Leu33^+^ cells were rare in the IgM^+^ IgD^−^ fraction, IgD expression seemed to depend on the affinity of IgM BCRs.

### IgM^+^ GC B cells differentiate into MBCs but not into plasmablasts

**Figure 2:**
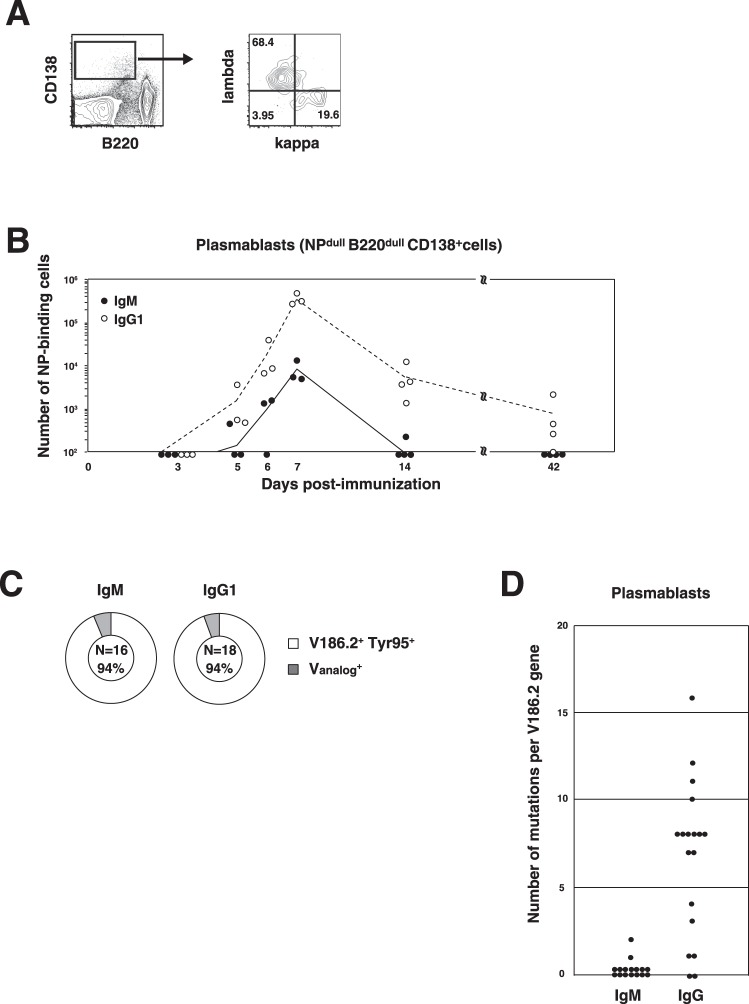
Comparison of the cell number, V_H_ usage, and SHM frequency between IgM^+^ and IgG1^+^ plasmablasts residing in spleens during Phase I and Phase II. (**A**) Flow cytometry of B220^−^ CD138^+^ cells in the spleen on day 7 postimmunization with NP_40_-CGG/alum. The cells were separated based on the expression of Igκ and Igλ. The numbers in the outlined areas indicate the percentages of Igκ^+^ Igλ^−^ cells (bottom right), Igκ^−^ Igλ^+^ cells (top left) and Igκ^−^ Igλ^−^ cells (bottom left). (**B**) Kinetic analysis of the number of IgM^+^ or IgG1^+^ plasmablasts with time. (**C**) Comparison of V_H_ usage between IgM^+^ and IgG1^+^ plasmablasts on day 7. (**D**) Comparison of the SHM frequency per *V186.2* gene between IgM^+^ and IgG1^+^ plasmablasts in the spleen on day 42 postimmunization. Data were pooled from four independent experiments with one mouse per experiment in Figure (**B**). Data are from 3–4 independent experiments with one mouse per time point indicated in the figures for each experiment (**A**–**C**).

We previously developed a method for discriminating between plasmablasts and plasma cells, enabling us to examine these ASCs separately^14^. Because most CD138^+^ cells were found to be mIgλ, they were largely plasmablasts on day 7 (Fig. 2A). In fact, the ratio of plasma cells in the total ASC population on day 7 was less than 0.02 (data not shown). Both IgM^+^ and IgG1^+^ plasmablasts were observed on day 5 and reached maximum cell numbers of ~10^4^ for IgM^+^ cells and ~3 × 10^5^ for IgG1^+^ on day 7 (Fig. 2B).

During Phase II, the number of plasmablasts decreased (Fig. 2B). Judging from the rapid decrease in number by day 14, plasmablasts in Phase I were considered to be short-lived. V_H_ sequence analysis of Phase I plasmablasts revealed that both IgM and IgG1 Abs were encoded by germline V186.2^+^ Tyr95^+^ genes (Fig. 2C). We sorted these day-42 plasmablasts and analyzed V_H_ nucleotide sequences, which revealed that SHMs were essentially absent in IgM^+^ cells while multiple SHMs were observed in the counterpart IgG1^+^ cells (Fig. 2D), suggesting that IgM^+^ plasmablasts had not arisen from IgM^+^ GC B cells on day 42 because GC B cells at this time point had multiple SHMs^7^. The presence of only SHM^+^ IgM^+^ MBCs (Fig. 1E) but not SHM^+^ IgM^+^ plasmablasts (Fig. 2D) suggested that IgM^+^ GC B cells are capable of differentiating into MBCs but not into plasmablasts or plasma cells.

### Comparison of transcription factor expression in IgM^+^ and IgG1^+^ GC B cells

**Figure 3:**
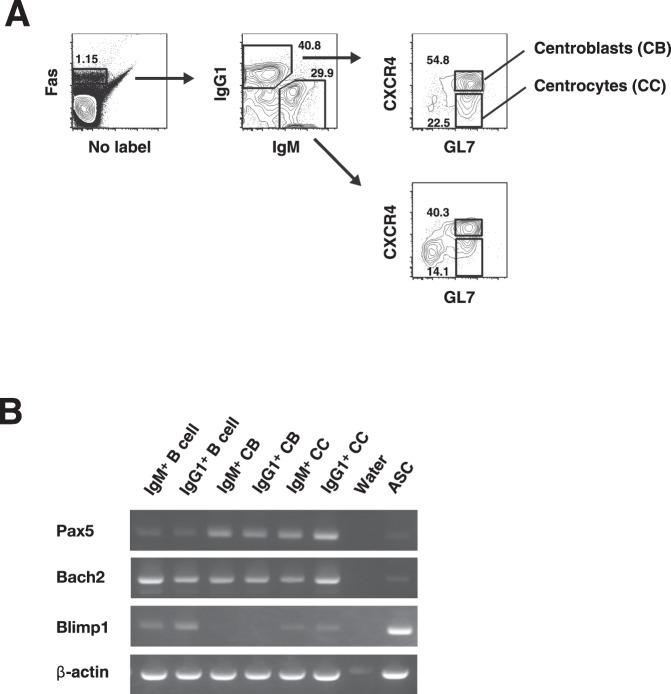
RT-PCR analysis of transcription factors expressed in IgM^+^ and IgG1^+^ centroblasts and centrocytes. (**A**) Isolation of centroblasts and centrocytes with IgM or IgG1 BCRs was performed on the basis of the expression of CXCR4 and GL7. (**B**) RT-PCR analysis of *Pax5* (Pax5), *Bach2* (Bach2), and *Prdm1* (Blimp1) mRNA expression between IgM^+^ and IgG1^+^ centroblasts or centrocytes obtained from spleens at 42 days postimmunization with NP_40_-CGG/alum. Data are representative of three independent experiments with one mouse per experiment.

We next compared the expression of transcription factors of IgM^+^ and IgG1^+^ GC B cells to determine whether the defect in IgM^+^ GC B cells in terms of their inability to differentiate into ASCs would relate to the expression of these factors. Blimp1 is the key regulator of ASC development, directly represses Pax5 and is negatively regulated by Bach2^20^. We isolated IgG1^+^ and IgM^+^ GC B cells on the basis of Fas expression, separated centroblasts and centrocytes based on the expression of CXCR4 and GL7 (Fig. 3A) and examined the expression of each transcription factor by RT-PCR (Fig. 3B). The results showed that IgM^+^ centrocytes expressed *Prdm1* mRNA at the same level as their IgG1^+^ counterparts and that there were no differences in *Pax5* or *Bach2* mRNA expression between these cells. Therefore, the defect in IgM^+^ GC B cells in differentiation into plasmablasts and plasma cells could not be explained in terms of transcription factor expression.

### Self-renewal and differentiation potential of MBCs after secondary immunization

**Figure 4:**
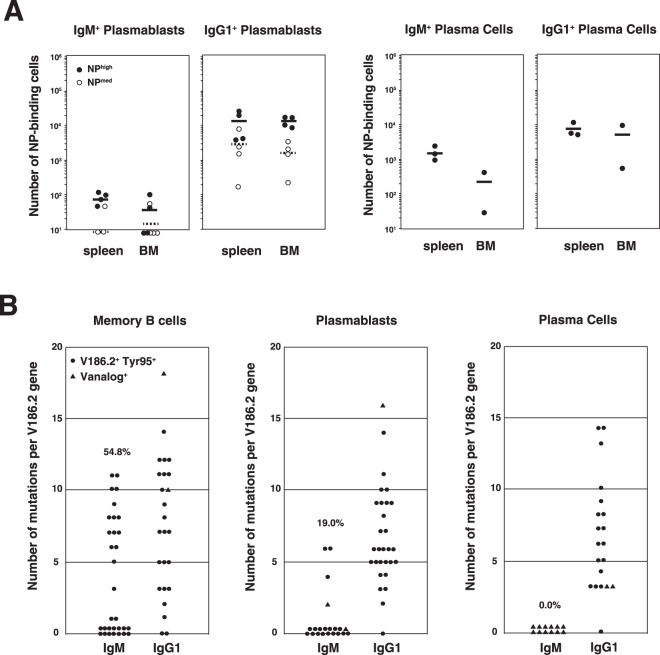
Comparative analysis of MBCs, plasmablasts, and plasma cells in Phase II. (**A**) Comparison of the numbers of IgM^+^ and IgG1^+^ plasmablasts and plasma cells in the spleen and bone marrow 6 weeks postimmunization. Closed circles (individual mice) and solid lines (averaged values) represent NP_hi_^+^ cells. Open circles (individual mice) and dotted lines (averaged values) pertain to NP_med_^+^ cells. (**B**) SHM frequency in V_H_ sequences of IgM^+^ and IgG1^+^ MBCs in the spleen and in plasmablasts and plasma cells in the spleen and bone marrow on day 42. Each dot represents an NP-specific clone: circle, V186.2; triangle, V_analog_. The number represents the frequency of SHMs per V_H_ gene. Data were pooled from 3–4 independent experiments with one mouse per experiment in (**A**). Data are from 3–4 independent experiments with one mouse per experiment.

Although IgM^+^ plasmablasts and plasma cells were nearly below the detection limit in Phase II (Fig. 2B), they appeared in the spleen and bone marrow after secondary immunization (Fig. 4A). In Phase III, the increase in the number of plasma cells was remarkable, as shown in a previous report^14^, which is true for IgM^+^ cells. We first examined which cells, MBCs or plasmablasts, were responsible for the generation of plasma cells, since both MBCs and plasmablasts express BCRs capable of receiving antigenic stimulation and since plasmablasts are thought to be precursors of plasma cells^21,22^. The results clearly showed that only transferred MBCs, and not plasmablasts, provided plasma cells that secreted anti-NP Abs (Fig. 4C). Although these experiments were carried out using plasmablasts regardless of the BCR isotype since few IgM^+^ plasmablasts were available, the results indicated that plasmablasts were unable to differentiate into plasma cells by secondary immunization with NP_40_-CGG.

Nucleotide sequence analysis revealed again that most of the IgM Abs secreted from plasmablasts and plasma cells had few SHMs (Fig. S1). This was even more evident in the V_H_ sequences of IgM^+^ plasma cells that secreted only V186.2^−^ SHM^−^ Abs. Taken together with the data showing that the number of plasmablasts was low compared with the number of plasma cells, these results suggested that IgM^+^ MBCs^non-GC^ had differentiated into plasma cells that secrete low-affinity IgM Abs after secondary immunization. In other words, IgM^+^ MBCs^GC^ were hardly differentiated into ASCs. This inability to differentiate into ASCs was not due to inefficient signaling through BCR since the V_H_ sequence analysis revealed that both IgM^+^ primary MBCs^non-GC^ and MBCs^GC^ were able to proliferate and self-renew by antigen stimulation, similar to IgG1^+^ MBCs (Fig. 4B).

### Isotype-dependent variation in Ab production and affinity with time during immunization

**Figure 5:**
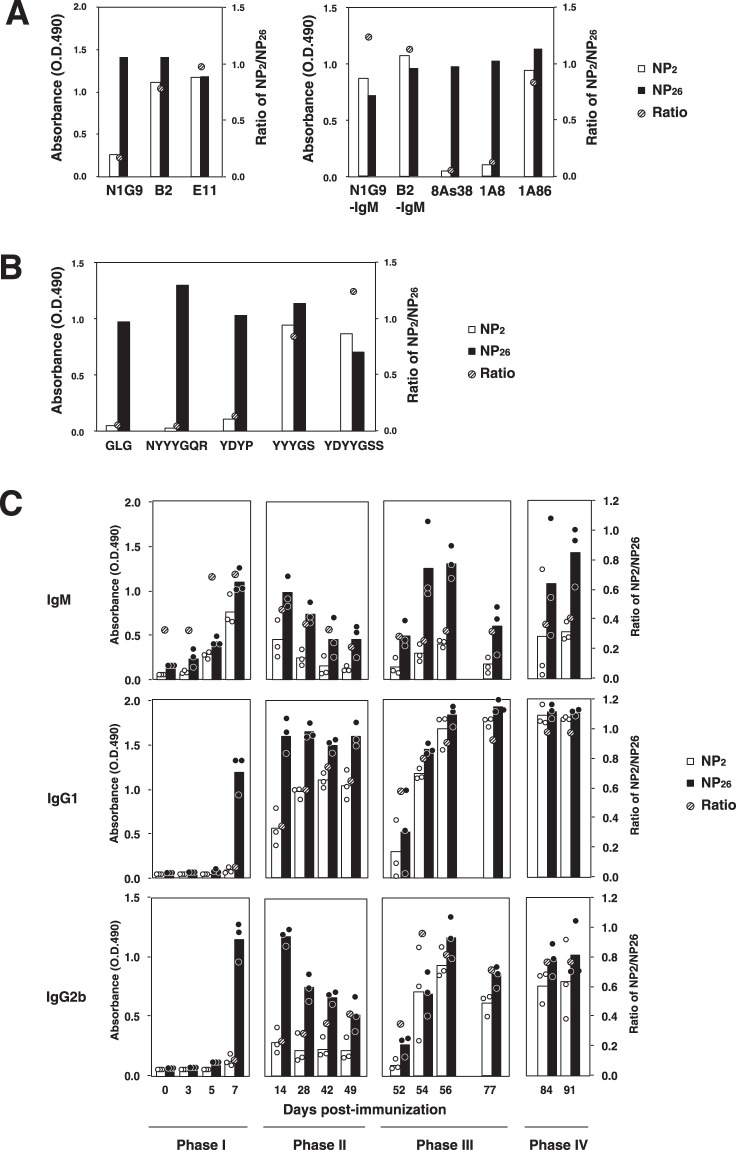
Changes in the amount of Ab bound to NP_2_-BSA or NP_26_-BSA and the NP_2_/NP_26_ ratio after immunization of C57BL/6 mice with NP_40_-CGG/alum. (**A**) Binding of IgG and IgM Abs to NP_2_-BSA (open bars) and NP_26_-BSA (closed bars). NP_2_/NP_26_ ratios are represented by hatched circles. Left panel, IgG mAbs with different Ka values to NPs, namely, N1G9 (Ka = 5 × 10^5^ M^−1^), B2 (Ka = 3.4 × 10^6^ M^−1^), and E11 (Ka = 10^8^ M^−1^), were used. Right panel, IgM mAbs secreted from hybridomas (8As38, 1A8, and 1A86, respectively) as well as recombinant IgM mAbs whose V-D-J genes were from N1G9 and B2 (N1G9-IgM and B2-IgM, respectively) were used. (**B**) Binding of IgM mAbs to NP_2_-BSA (open bars) and NP_26_-BSA (closed bars). NP_2_/NP_26_ ratios are represented by hatched circles. The CDR3 sequences of the IgM mAbs are shown. (**C**) Changes in the binding of IgM and IgG2b Abs in the immune sera. The serum from an immunized mouse (n = 3) was diluted (200-fold for IgM, 800-fold for IgG2b), and binding was measured by ELISA. Open circles (individual mice) and open bars (averaged values) represent binding to NP_2_-BSA. Closed circles (individual mice) and closed bars (averaged values) represent binding to NP_26_-BSA. The time course of Ab production was divided into four periods: Phase I, Phase II, Phase III and Phase IV. Phase IV corresponds to the period post tertiary immunization. Data are representative of three independent experiments per experiment (**A**,**B**) or three independent experiments with one mouse per experiment (**C**).

**Figure 6:**
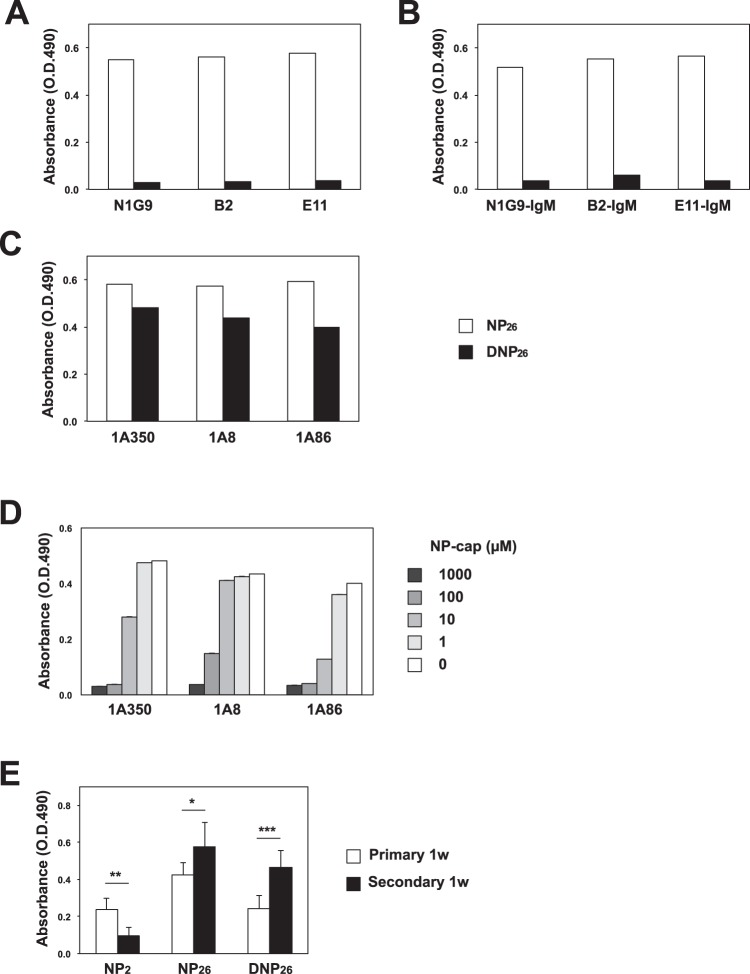
NP-binding IgM Abs possess cross-reactivity to analogs of the original antigen. (**A**) Binding of IgG mAbs to NP_26_-BSA (open bars) and DNP_26_-BSA (closed bars). IgG mAbs with different Ka values to NPs, namely, N1G9 (Ka = 5 × 10^5^ M^−1^), B2 (Ka = 3.4 × 10^6^ M^−1^), and E11 (Ka = 10^8^ M^−1^), were used. (**B**) Binding of IgM mAbs to NP_26_-BSA (open bars) and DNP_26_-BSA (closed bars). Recombinant IgM mAbs whose V-D-J genes were from N1G9, B2 and E11 (N1G9-, B2- and E11-IgM, respectively) were used. (**C**) Binding of IgM mAbs secreted by hybridomas (1A350, 1A8, and 1A86, respectively) to NP_26_-BSA (open bars) and DNP_26_-BSA (closed bars). (**D**) Binding of IgM mAbs performed as in (**B**) in the presence of the indicated concentrations of NP-Cap. (**E**) Binding of IgM Abs to NP_2_-, NP_26_-, and DNP_26_-BSA in serum collected from an individual mouse (n = 3) at 1 week after primary immunization (primary 1w) and secondary immunization (secondary 1w). The serum was diluted 200-fold, and binding was measured by ELISA. *p < 0.05, **p < 0.01, and ***p < 0.001. Error bars indicates the s.d. Data are representative of three independent experiments per experiment (**A**–**D**) or four independent experiments with one mouse per experiment (**C**).

Ab affinities are often estimated by ELISA using hapten-protein conjugates with different numbers of hapten molecules, such as NP_2_-BSA and NP_26_-BSA. In the case of germline IgG Abs (V186.2^+^ Tyr95^+^ Trp33^+^), Abs such as N1G9 cannot bind to NP_2_-BSA, but affinity-matured IgG Abs such as B2 (V186.2^+^ Tyr95^+^ Leu33^+^) or E11 (V186.2^+^ Gly95^+^ SHM^+^) can (Fig. 5A). On the other hand, IgM Ab has a pentameric structure and thus possesses ten antigen-combining sites per molecule, which allows binding to multivalent antigens with higher avidity than that of IgG Abs, as shown with control IgM Abs encoded by the same V_H_ and V_L_ genes as IgG counterparts, N1G9 and B2 (Fig. 6A). From the viewpoint of the *V*_*H*_*-D-J*_*H*_ gene nucleotide sequence, N1G9-IgM is identical to B1-8^18,23^. On the other hand, some IgM Abs, such as 8A38 and 1A8^10^, which are V186.2^+^ X95^+^, are unable to bind to NP_2_-BSA, and their NP_2_/NP_26_ ratios are lower than that of N1G9-IgM (Fig. 5A), suggesting that they have very low affinities. In fact, they are unable to bind to NP_26_-BSA when converted into the IgG form (Murakami *et al*., unpublished observation). Since these IgM Abs are encoded by *V186*.*2* genes with no SHMs^10^, their very low affinities are considered to arise from differences in CDR3H sequences (Fig. 5B).

Next, we examined the binding of IgM Abs to NP_2_- and NP_26_-BSA in immune sera obtained from mice immunized with NP_40_-CGG in Phase II (Fig. 5C). The total amount of IgM Ab bound to NP_26_-BSA was estimated with the OD_490_ value, which decreased steadily with time until day 49, indicating that there was a short supply of IgM Abs in Phase II. On the other hand, a large amount of Ab production was observed on day 54 or 56 after secondary immunization (Phase III), indicating that recall Ab production was induced. However, IgM Abs on day 56 showed inefficient binding to NP_2_-BSA and provided a low NP_2_/NP_26_ ratio of ~0.2–0.3, which was smaller than that of day 7 Abs (~0.7), suggesting that the secondary Abs have lower affinity (avidity) than the primary Abs. The lower NP_2_/NP_26_ ratio for day 56 IgM Abs was not due to inhibition by the binding of high-affinity IgG Abs to NP_2_-BSA since similar results were obtained using antisera from which IgG Abs had been removed with protein G-beads (Fig. S2A).

An Ab production profile that was different from that of IgG1 Abs was observed for other isotypes. The peak of primary IgG2b Ab production was detected at approximately 7–14 days, followed by a gradual decrease with time. The ratio of NP_2_/NP_26_ increased slightly (0.2–0.4), but the decrease was not as evident as that of IgG1 Abs. Secondary immunization, however, resulted in an obvious increase in the NP_2_/NP_26_ ratio (0.9–1.0), indicating that the recall response of IgG2b Abs was accompanied by the production of affinity-matured Abs (Fig. 5D). We also examined the production of IgG3 and IgA Abs (Fig. S3). The peak production of IgG3 Abs was observed at approximately 7–14 days, followed by a gradual decrease during Phase II, with little change in the NP_2_/NP_26_ ratio, which was as low as <0.2. In the case of IgA Abs, neither a production peak at approximately day 7 nor subsequent Ab production in Phase II was observed, indicating that the primary response of these Abs was different from those of the others. In conclusion, these results show that each isotype has a unique Ab production profile and may not necessarily be accompanied by an increase in Ab affinity (Figs 5C and S3).

### Cross-reactive binding of secondary IgM Abs to DNP-hapten

To understand the immunological role of secondary IgM Abs, we examined their cross-reactivity to various chemical compounds, since cross-reactions sometimes play an important role in viral infection^24^. Anti-NP Abs are known to react with NP analogs, such as (4-hydroxy-3-iodo-5-nitrophenyl)acetyl (NIP) or (4-hydroxy-3,5-dinitrophenyl)acetyl (NNP), which are haptens with a higher affinity than that of NP alone; this phenomenon is referred to as heterocliticity^25,26^. We also examined whether anti-NP IgG1 Abs can bind to DNP_26_-BSA and found that they are unable to bind to DNP regardless of their affinity (Fig. 6A), even though the structures of the IgG Abs were converted to the pentameric IgM form (Fig. 6B). On the other hand, the IgM Abs 1A350, 1A8, and 1A86, which are encoded by germline *V186*.*2* genes^10^, could bind to DNP_26_-BSA (Fig. 6C). Such cross-reactive binding to DNP would not be caused by a nonspecific hydrophobic interaction since the Abs did not bind to (4-hydroxyphenyl)acetyl (HP)_26_-BSA, FITC-BSA or acetyl (Ac)-BSA (Table 1). In addition, this binding was inhibited with NP-Cap in a dose-dependent manner, suggesting that the same binding sites were recognized by both NP and DNP (Fig. 6D).

**Table 1:** Cross-reactions with hapten-BSAs.

	NP_26_	NP_2_	DNP_26_	TNP_26_	HP_26_	Ac	FITC
N1G9	++	−	−	−	−	−	−
B2	++	++	−	−	−	−	−
E11	++	++	−	−	−	−	−
N1G9-IgM	++	++	−	−	−	−	−
B2-IgM	++	++	−	−	−	−	−
1A350	++	++	++	+	−	−	−
1A8	++	−	++	−	−	−	−
1A86	++	++	++	−	−	−	−
8As38	++	−	−	−	−	−	−

++0.5>, +0.5~0.05, −0.05 < (O.D. 490).

We also compared the binding of IgM Abs to NP_2_, NP_26_, and DNP_26_-BSA using NP-CGG immune sera (Fig. 6E). Day 7 IgM Abs showed efficient binding to NP_2_-BSA, while day 56 IgM Abs showed weak binding to NP_2_-BSA but significant binding to DNP_26_-BSA and to NP_26_-BSA (Fig. 6E). In terms of NP/DNP cross-reactivity at the molecular level, the appearance of Igλ^+^ NP^+^ DNP^+^ MBCs supported these observations (Fig. S4).

## Discussion

In this study, we analyzed IgM^+^ MBCs phenotypically and functionally based on our previous reports^7,10^, focusing on IgM^+^ ASCs and Abs secreted from these cells. Subcategorizing IgM^+^ MBCs on the basis of their expression of IgD BCR, we found that the SHM^+^ and SHM^−^ fractions corresponded to IgM^+^ IgD^−^ and IgM^+^ IgD^+^ cells, respectively. Although these two types of IgM^+^ MBCs possessed the same memory characteristic of self-renewal potential, there was a difference in the ability to differentiate into ASCs; IgM^+^ IgD^+^ MBCs, but not IgM^+^ IgD^−^ MBCs, showed poor potential for ASC formation, in contrast to classical views on memory definition^8,9^. Notably, our study revealed that secondary IgM Abs exhibited cross-reactivity, while their affinities were lower than those of canonical IgM Abs with strict antigen specificity. In contrast, Krishnamurty *et al*. reported that IgM^+^ IgD^−^, but not IgM^+^ IgD^+^ MBCs, were highly mutated in the malaria immunization system^27^. However, since Pape *et al*.^28^ demonstrated that IgM^+^ IgD^+^ MBCs in the PE/CFA immunization system dominantly comprised those possessing fewer SHMs, IgM^+^ IgD^+^ MBCs likely expressed few SHMs, and the discrepancy may be explained in terms of antigens employed, namely, NP, PE or malaria. Obviously, malaria-antigen epitopes would be more complex than NP and PE and activate more diverse B cell repertoires with a broad range of affinities to be involved in Ab production. This brought about the enrichment of heterogeneous MBCs due to wider gating of antigen-specific populations and a broader range for *Ig* gene sequence analysis.

Our results showing that IgM^+^ MBCs^non-GC^ with low-affinity BCRs are able to differentiate into ASCs responsible for recall IgM Ab production are not consistent with the notion that high-affinity B cells are selected to differentiate into plasma cells^14,29^. We hypothesized that high-affinity IgG1 Abs may prevent the secretion of IgM Abs, as reported for TI antigens^30^; however, no such effect of IgG1 Ab on the recall response of IgM Abs was observed (Fig. S4). Therefore, it is likely that a lack of differentiation of IgM^+^ MBC^GC^ into ASCs might be caused by isotype-specific and affinity-dependent regulation of BCR signaling^31–34^. Our finding that the two IgM^+^ MBC subsets seemed to be distinguishable on the basis of IgD BCR expression (Fig. 1E) will be useful to elucidate the underlying mechanisms.

In addition to MBCs, as described above, a rapid decline in the number of IgM^+^ plasmablasts indicated poor potential to differentiate into ASCs among IgM^+^ GC B cells (Fig. 2B). Furthermore, most of the IgM Abs secreted from plasmablasts on day 42 had no SHMs (Fig. 2D). These results indicated the preference for skewed MBC differentiation in IgM^+^ GC B cells in contrast to IgG1^+^ GC B cells. Surprisingly, the inability of IgM^+^ GC B cells to differentiate into plasmablasts was not due to insufficient expression of transcription factors, since no significant differences in the levels of transcription factors were observed between IgM^+^ and IgG1^+^ GC B cells (Fig. 3). Considering the small difference in BCR affinity between IgM^+^ and IgG1^+^ GC B cells^7^, we supposed that IgM^+^ GC B cells were prohibited from differentiating into plasmablasts capable of secreting high-affinity IgM Abs that are inferior to low-affinity Abs in such functions as complement-dependent hemolysis^10^, although these cells were allowed to differentiate into MBCs, whose regulatory mechanism has not yet been revealed.

During Phase III, a large amount of IgM Ab capable of binding to NP_26_-BSA, but not to NP_2_-BSA, was secreted. Although both Phase I- and Phase III-IgM Abs lack SHMs, they are different in terms of their initial affinities (Fig. 5C). Despite their low affinities, secondary IgM Abs have a unique antigen specificity that makes them capable of binding to DNP and TNP; no other anti-NP Abs, including primary IgM Abs and affinity-matured secondary IgG1 Abs, showed any ability to bind to NP analogs (Fig. 6 and data not shown). We suppose that the immune system prepares two different types of IgM Abs in terms of antigen recognition in Phases I and III, which conclude on days 7 and 54, respectively (Fig. 5). Although the intrinsic affinities of the combining sites are similar, IgM Abs have the advantage over IgG1 Abs of being able to bind to multivalent antigens through their higher avidity because the IgM Abs have 10 combining sites per molecule, suggesting that primary IgM Abs play a role in compensating for the low affinities of IgG1 Abs. Secondary immunization also provides both IgM and IgG1 Abs, whose affinities are significantly differ (Fig. 5C). Since affinity maturation has been shown to accompany specificity maturation^35^, affinity-matured IgG1 Abs recognize antigens more efficiently and more precisely than their precursors. However, microbes that invade our bodies sometimes change their surface structures to escape attack from affinity-matured IgG1 Abs. Immune systems seem to prepare IgM Abs with a broad specificity, which is expected to extend beyond the narrow specificity of IgG1 Abs and function as a first-line barrier against the invasion of microbes whose epitopes are altered by mutations through an Ab cross-reaction mechanism. Krishnamurty *et al*. provided new knowledge on IgM^+^ MBCs and ASCs by utilizing the malaria immunization system^27^. Tracing the changes in the SHM frequencies, affinities and specificities of IgM Abs through comparative analysis of the changes in those of IgG Abs in a clinically relevant setting is informative.

Classically, MBCs are defined as cells that have undergone antigen-driven proliferation, expressed isotype-switched BCRs, and then become resting cells. They are activated upon re-exposure to primed antigens and then proliferate and subsequently differentiate into ASCs capable of secreting high-affinity Abs^8,9^. These types of MBCs are sometimes referred to as effector MBCs, and IgG1^+^ MBCs^GC^ meet this definition. Judging from the profiles of Ab production and affinity maturation, IgG2b^+^ MBCs would be characterized as the effector type (Fig. 6), although their frequency is quite low during late Phase II at both the cellular level (IgM^+^, 63.3 ± 7.0%; IgG1^+^, 28.5 ± 6.3%; IgG2b^+^, 2.2 ± 0.8% in NP_hi_^+^ B cells) and sequence level^7^. On the other hand, IgM^+^ MBCs are referred to as central MBCs because they lack the ability to differentiate into ASCs but are able to re-enter into GCs and differentiate into IgG^+^ MBCs^12,36^. However, since we found that IgM^+^ MBCs^non-GC^ have the potential to differentiate into plasma cells that secrete secondary IgM Abs, they can be referred to as the effector type. Zuccarino-Catania *et al*. recently demonstrated remarkable data that effector MBCs and central MBCs were distinguished by the expression of CD80 and PD-L2^37^. Further characterization of IgM^+^ MBCs^non-GC^ with the expression patterns of surface markers^12,37^ and transcriptional factors^20^ is expected. Although little information is available regarding their function of IgM^+^ MBCs^GC^, we speculate that IgM^+^ MBCs^GC^ may be a new type of MBC possessing affinity-matured BCRs capable of switching to other isotypes when necessary. The immunological role of IgM^+^ MBCs^GC^ needs to be clarified as well as that of MBCs with isotypes other than IgG1.

In a series of reports^6,7,14^, we have described the basis of the cellular dynamism of Ab production and affinity change and showed divergent and unique patterns that are specific to such isotypes as IgG1, IgG2b, and IgM. Vaccines have been used to activate naïve B cells to differentiate into MBCs and ASCs and are expected to prevent the invasion of microbes, which are known to stimulate the production of various Ab isotypes. For efficient and safe vaccination, further knowledge on the regulation of the MBCs and ASCs of each isotype is required.

## Methods

### Mice and Immunizations

C57BL/6 mice were purchased from Japan SLC. AID^−/−^ mice were provided by Prof. T. Honjo (Kyoto University, Kyoto, Japan). All mice were maintained in Tokyo University of Science (TUS) mouse facility under specific pathogen-free conditions. Mouse procedures were performed under protocols approved by the TUS Animal Care and Use Committee and are in accordance with the current national regulation. Mice were immunized i.p. with 100 μg of NP_40_-CGG precipitated in alum and boosted with the same Ag in PBS.

### NP-Protein Conjugates

NP-protein conjugates were prepared as described previously^38^. Briefly, the hydroxysuccinimide ester of NP (NP-OSu) was coupled with proteins in 0.1 M sodium bicarbonate buffer (pH 8.5) containing 0.15 M NaCl for 1 h at room temperature. Any excess of reagents or reaction byproducts was removed via passage through a Sephadex G-25 column (GE Healthcare) or by dialysis against PBS. In the case of NP-allophycocyanin (NP-APC) and streptavidin-NP-APC (NP-APC_avi_), the number of NPs per APC was assumed on the basis of the molar ratio of NP-Osu to APC or APC_avi_ in the reaction mixture^6^. DNP- and TNP-BSA were prepared by reacting BSA with 2,4-dinitrobenzenesulfonic acid or 2,4,6-trinitrobenzenesulfonic acid^39^. HP-BSA was prepared by reacting BSA with the hydroxysuccinimide ester of HP. The number of HPs introduced was estimated by titration of amino groups with 2,4,6-trinitrobenzenesulfonic acid^40^.

### Anti-NP Abs

Hybridomas producing the anti-NP Abs 8A38, 1A8, 1A86, 9T13, B2 and E11 were prepared as described previously^5,10,38^. N1G9 was provided by Prof. K. Rajewsky (Max-Delbrück-Centrum für Molekulare Medizin, Berlin, Germany). The V_H_-D_H_-J_H_ genes of N1G9 or B2 were fused with the secreted form of Cμ-genes and transfected into J558L myeloma cells by electroporation. N1G9-IgM and B2-IgM were purified from culture supernatants^6^.

### Flow Cytometry and Detection Reagents

Cells were treated with 5 mM EDTA in PBS before staining, which reduced the binding of cytophilic nonendogenous Abs to B cells (Supplemental information). Cells were treated with unlabeled anti-CD16/CD32 (2.4G2, eBioscience) in FACS buffer (1% BSA/PBS) to block FcγRs. After washing, cells were stained with the relevant antibodies: anti-Igκ-FITC (187.1, BD Pharmingen), anti-IgM-PerCP-Cy5.5 (II/41, eBioscience), anti-IgD-FITC (11–26 c.2a, BD Pharmingen), anti-IgG1-PE (A85-1, eBioscience), anti-CD45R/B220-PE-Cy7 (RA3-6B2, eBioscience), anti-CD138-Biotin (281.2, BD Pharmingen), anti-GL7-FITC (GL7, BD Pharmingen), anti-CD38-PE (90, eBioscience), anti-CD95/Fas-PE (Jo2, BD Pharmingen), anti-CXCR4-APC (2B11, eBioscience), and PE–streptavidin (eBioscience). Cells were analyzed on FACSCanto II and FACSAria II (BD Biosciences). Cell sorting was performed on FACSAria II (BD Biosciences). The data were analyzed using FlowJo software (Tree Star).

### Detection of Cells Secreting Antibodies by Flow Cytometry

We employed the method as described previously^14^. Cells were treated with unlabeled rat anti-mouse CD16/CD32 (2.4G2, eBioscience) in medium (10% FCS/IMDM) to block FcγRs. After washing with medium, cells were stained with rat anti-Igλ-PE (JC5-1, Southern Biotech) and biotinylated rat anti-mouse CD138 (281-2, BD Pharmingen) in medium. FACS buffer (1% BSA/PBS) containing no azide was used for washing cells and subsequent staining with streptavidin-APC (BioLegend) coupled with NP. After cultivation, the cells were washed and stained in FACS buffer containing sodium azide. Finally, cells were stained with rat anti-Igλ-FITC (JC5-1, Southern Biotech) and rat anti-mouse B220 (RA3-6B2, eBioscience). Cell sorting was carried out using a FACSAriaII (BD Biosciences). Files were analyzed using FlowJo software (Tree Star).

### V_H_ Sequence Analysis

mRNAs were purified from sorted cells of each B cell subset using Trizol (Invitrogen). cDNAs were synthesized using PrimeScript Reverse Transcriptase (Takara) and Oligo-dT primers, followed by amplification using PrimeSTAR DNA polymerase (Takara) and forward primer: 5′-TCTAGAATTCAGGTCCAACTGCAGCAGCC-3′, paired with the following reverse primers: *mCμ*5′-CTTGAACAGGGTGACGG-3′ for IgM^+^ MBCs, *sCμ*5′-GTGTCAGACATGATCAGG-3′ for IgM^+^ plasmablasts/plasma cells; *mCγ*5′-TCTGCTTCAGCTCCACCAC-3′ for IgG^+^ MBCs; *sCγ1* 5′-TCTGCTTCAGCTCCACCAC-3′ and *sCγ2b/2c* 5′-GTGCTGAGCTCATTTACC-3′ for IgG^+^ plasmablasts/plasma cells.

### Transcriptional Factor Expression Analysis

mRNAs were purified from 1,000 sorted cells of each B cell subset using Trizol (Invitrogen). cDNAs were synthesized using PrimeScript Reverse Transcriptase (Takara) with random primers. PCR was performed using *Takara* Ex Taq HS (Takara) and the following primers^41–43^: Pax5 5′-CCTACCCTATTGTCACAGGCC-3′, 5′-CCTCTGTCTGTCTCAGGGGGTT-3′; Bach2 5′-GCTGCTCCACACTGTGACAT-3′, 5′-ACCTCCCGGATGTTCTCTCT-3′; and Blimp1 5′-AACACGTGGTACAACCCAAAG-3′, 5′-AGGCTGCAGAGATGGATGTAG- 3′.

### ELISA

To measure the binding of Abs to hapten, plates (Nunc) were coated with 50 μl per well of hapten-BSA in PBS and left at 4 °C overnight, followed by blocking with FACS buffer (1% BSA/0.05% sodium azide/PBS). After washing with PBS-Tween 20 (PBST), Abs of known concentrations or antisera at various dilutions were added, and the plates were then incubated for 2 h at room temperature. After washing with PBST, the bound Abs were detected with HRP-conjugated goat antibodies specific to each mouse isotype (Southern Biotech).

### Statistics

Statistical differences were analyzed with the 2-tailed Student’s t-test for 2-group comparisons and one-way ANOVA with Tukey’s test for multiple comparisons, with Prism 7 software (GraphPad Software, La Jolla, CA, USA). Data were considered significant at *p < 0.05, **p < 0.01, and ***p < 0.001.

## Electronic supplementary material

Supplemental Figures

## Data Availability

The datasets generated and/or analyzed during this study are available from the corresponding author on reasonable request.
