# Supplementary material for: High-affinity IgM+ memory B cells are defective in differentiation into IgM antibody-secreting cells by re-stimulation with a T cell-dependent antigen
Source: Sci Rep. 2018 Sep 28;8:14559. doi: 10.1038/s41598-018-32926-w (PMC6162211; doi:10.1038/s41598-018-32926-w)
Supplement: Supplementary file 1 — Supplemental Figures [file 41598_2018_32926_MOESM1_ESM.pdf]

Supplemental Figures

High-affinity IgM+ memory B cells are defective in differentiation into IgM antibody-secreting cells by re-stimulation with a T cell-dependent antigen

Yasuyuki Tashiro[1,2,*], Akikazu Murakami[3], Yasushi Hara[4], Takeyuki Shimizu[5], Masato Kubo[6,7], Ryo Goitsuka[1], Hidehiro Kishimoto[3], and Takachika Azuma[2,8]

[1]Division of Development and Aging, [2]Division of Biosignaling, [4]Shared equipment room, [6]Division of Molecular Pathology, Research Institute for Biomedical Sciences, Tokyo University of Science, Noda, Chiba, Japan

[3]Department of Parasitology & Immunopathoetiology, Graduate School of Medicine, University of the Ryukyus, Okinawa, Japan

[5]Department of Immunology, Kochi Medical School, Kochi University, Kochi, Japan

[7]Laboratory for Cytokine Regulation, Research Center for Integrative Medical Science (IMS), RIKEN Yokohama Institute, Yokohama, Kanagawa, Japan

[8]Antibody Technology Research Center, Co. Ltd., Noda, Chiba, Japan

*Correspondence: ta.yasuyuki@gmail.com (Y. T.)

Figure S1 | Cell source to supply plasma cells at secondary response.

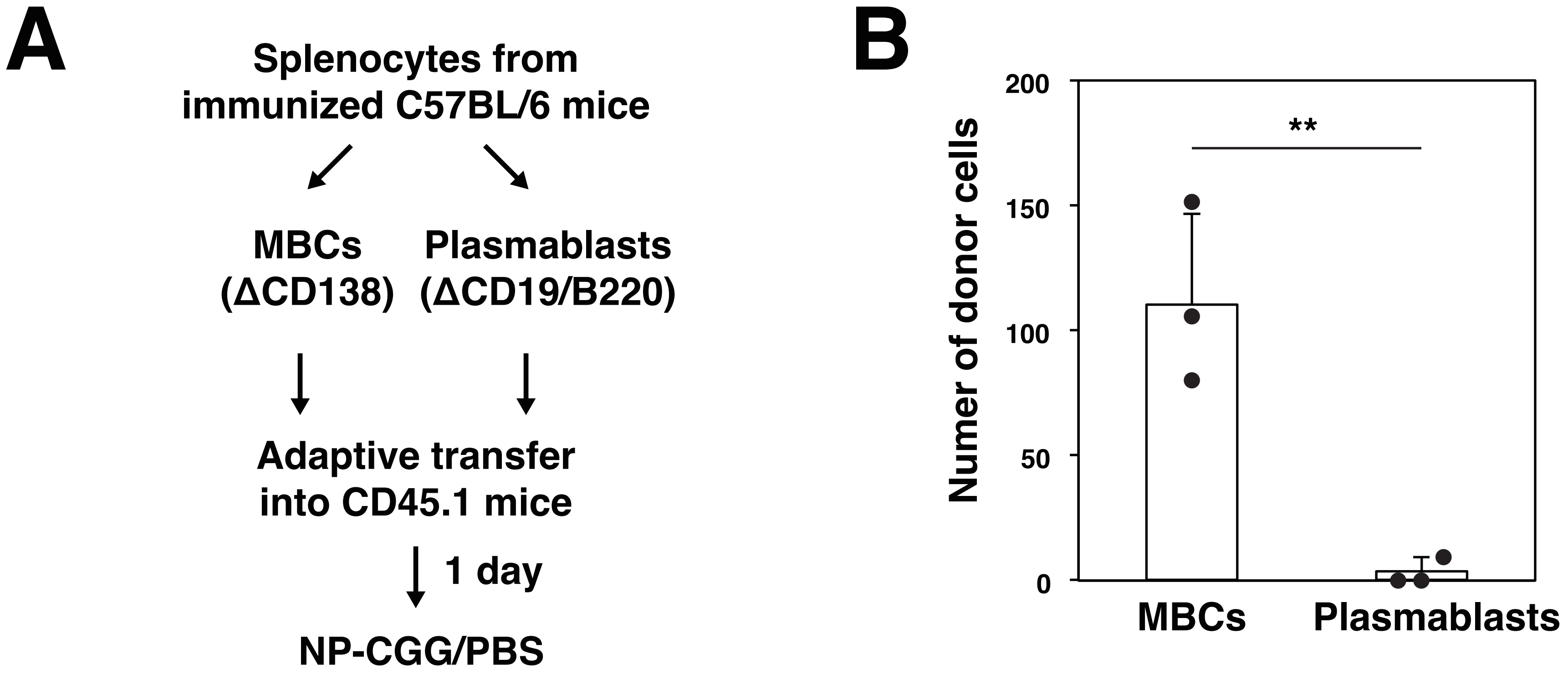

(A) Experimental design for evaluating the ability to develop into plasma cells using an adoptive transfer system. All spleen cells were pooled from two C57BL/6-CD45.2 mice on day 42 postimmunization with NP_{40}-CGG/alum. RBCs were lysed with ammonium chloride, and cells were divided into two fractions for equal numbers. Each fraction of cells was incubated with anti-CD138-Biotin (281-2, BD Pharmingen) for MBC transfer and anti-CD19-Biotin (1D3, BD Pharmingen) and anti-CD45R/B220-Biotin (RA3-6B2, eBioscience) for plasmablast transfer, followed by negative sorting using the MACS system (Miltenyi Biotec) and iMag system (BD Biosciences). C57BL/6-CD45.1 mice were i.v. administered the isolated cells one day before the secondary immunization with NP_{40}-CGG/PBS.

(B) C57BL/6-CD45.1 mice received MBCs (fraction depleted of $CD138^{+}$ cells) or plasmablasts (fraction depleted of $CD19^{+}$ $B220^{+}$ cells) prepared on day 42 from congenic $CD45.2^{+}$ mice that had been immunized with NP_{40}-CGG/alum. One day after adaptive transfer, the mice were immunized with NP_{40}-CGG/PBS, and the number of donor cells was analyzed 7 days after immunization. **$p < 0.01$.

Data are from three independent experiments with two mice (1 mouse for the MBC group and 1 mouse for the plasmablast group) per experiment.

Figure S2 l Effect of IgG Abs on IgM binding to NP-BSA.

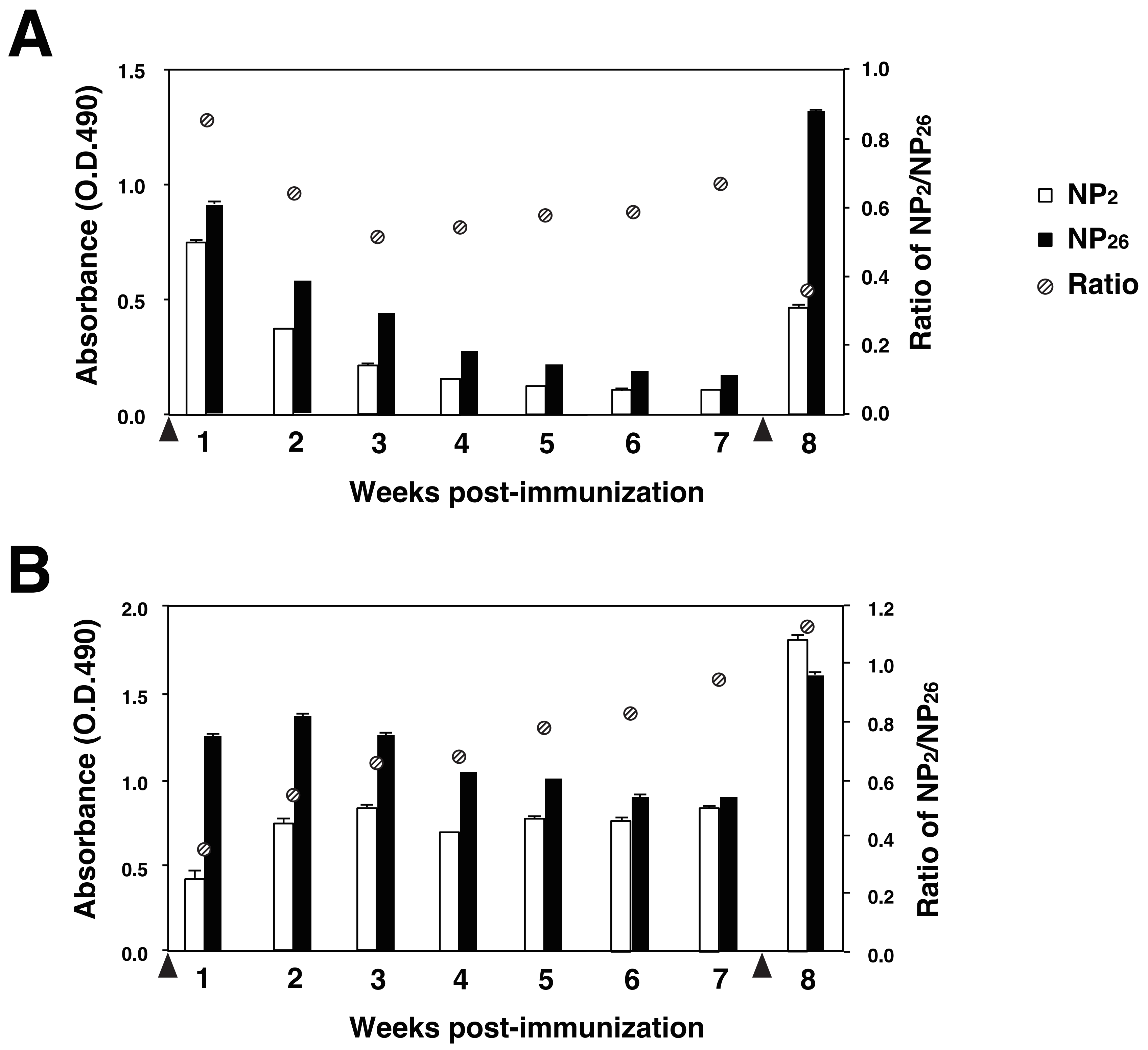

Binding of IgM and IgG Abs to NP_2-BSA (open bars) and NP_{26}-BSA (closed bars). NP_2/NP_{26} ratios are represented by hatched circles. The triangle shows the timing of secondary immunization with NP_{40}-CGG/PBS. (A) The serum from which IgG Abs were removed with protein G-beads was diluted 200-fold, and IgM Ab binding was measured by ELISA. (B) The serum that was not treated with protein G-beads was diluted 1,000-fold, and IgG Ab binding was measured by ELISA. Data are representative of three independent experiments per experiment.

Figure S3 | Changes in the amount of Ab bound to NP_2-BSA or NP_{26}-BSA and in the NP_2/NP_{26} ratio after immunization of C57BL/6 mice with NP_{40}-CGG/alum.

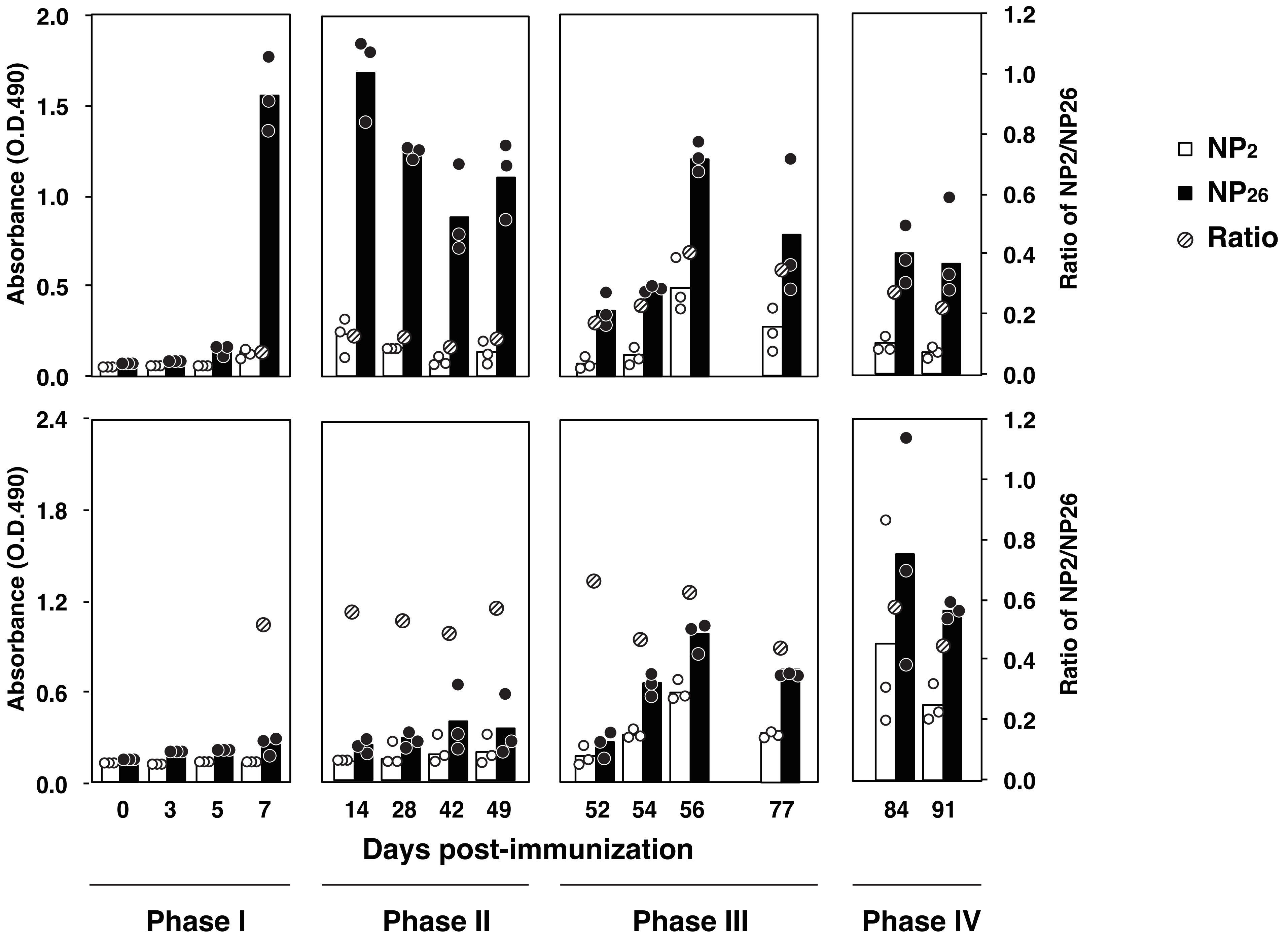

The serum from an individual immunized mouse (n = 3) was diluted (200-fold for IgG3 Abs and 200-fold for IgA Abs), and binding was measured by ELISA. Open circles (individual mice) and open bars (averaged values) represent binding to NP_2–BSA. Closed circles (individual mice) and closed bars (averaged values) represent binding to NP_{26}–BSA. The time course of Ab production was divided into four periods: Phase I, Phase II, Phase III, and Phase IV. Data are representative of three independent experiments with three mice per experiment.

Phase I: early and rapid production of low-affinity Abs on day 7. Phase II: continuous Ab production between days 14 and 49. Phase III: Ab production after secondary immunization. Phase IV: late Ab production after tertiary immunization.

Figure S4 | Analysis of the cell number of NP^{+} DNP^{+} B cells after immunization with NP_{40}-CGG.

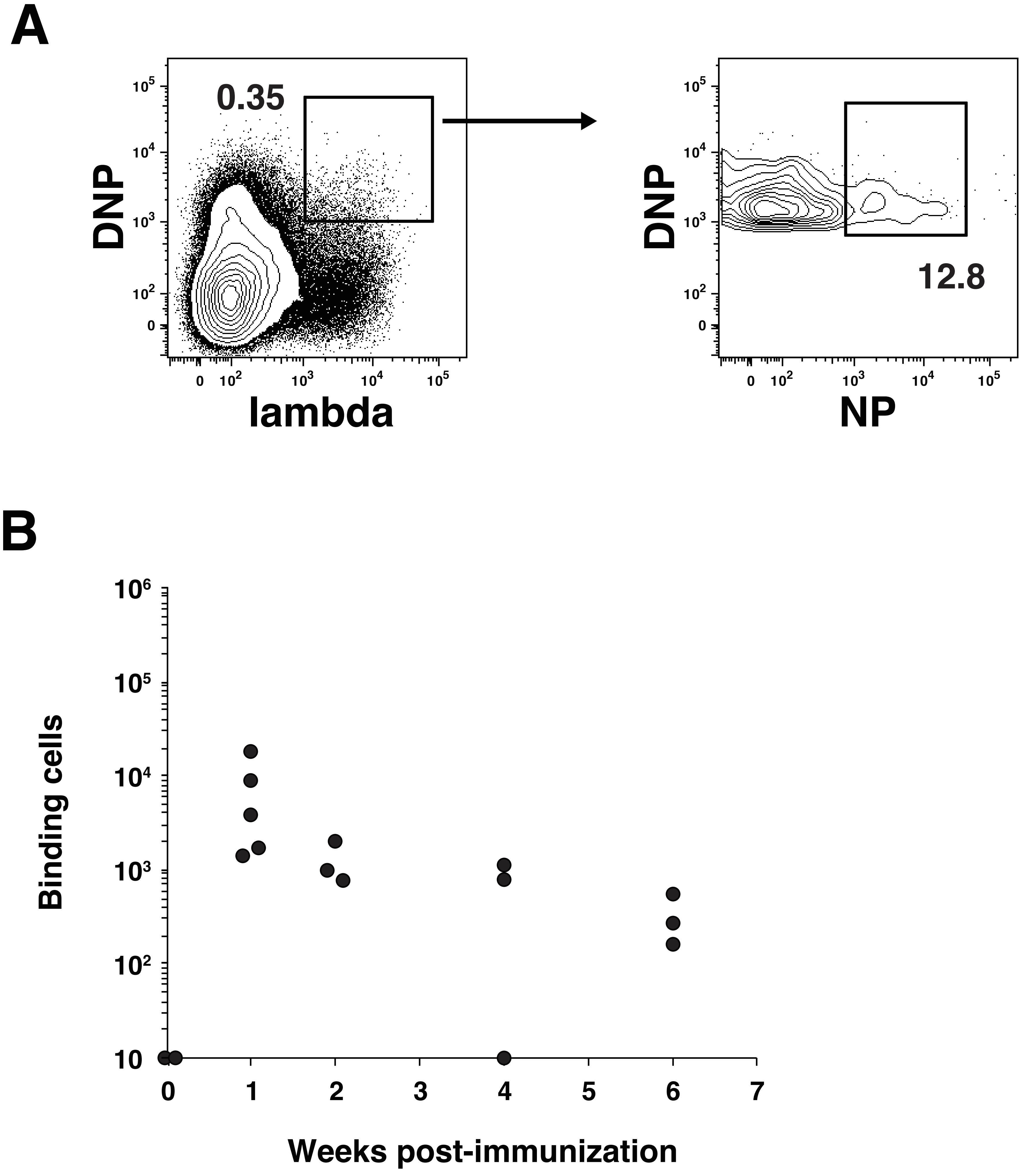

(A) Flow cytometry of B220^{+} Igλ^{+} cells in the spleen on day 7 postimmunization with NP_{40}-CGG/alum. The cells were separated based on binding to NP and DNP. (B) Kinetic analysis of the number of NP^{+} DNP^{+} B cells. Data are from 3-5 independent experiments with one mouse per time point indicated in the figures for each experiment.

Figure S5 l Effect of IgG Abs on the generation of high-affinity IgM Ab-secreting cells.

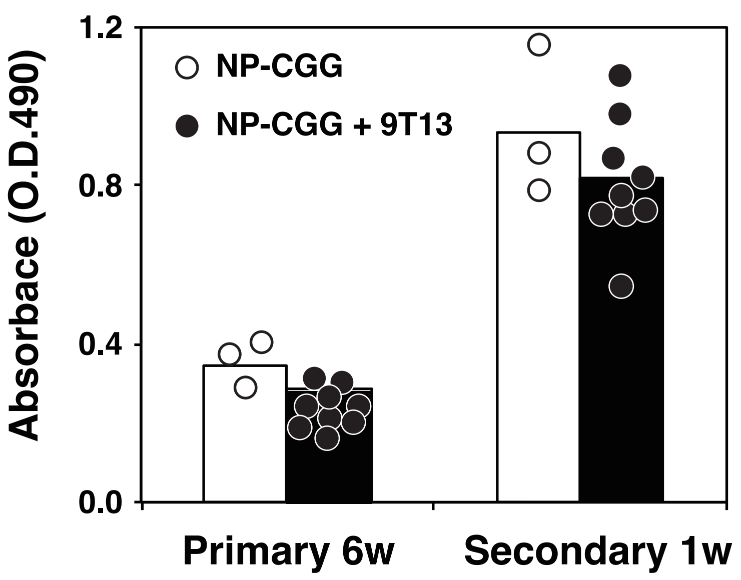

AID$^{-/-}$ mice immunized with NP_{40}-CGG/alum were intravenously injected with 9T13 (V186.2^{+} Gly95^{+} IgG1 mAbs) one day before secondary immunization. The serum was recovered one week before and after secondary immunization (primary 6w and secondary 1w, respectively). IgM binding was measured with 200-fold diluted serum by ELISA. Data are from three independent experiments with three mice (1 mouse in the control group; 2 mice in the 9T13 group) per time point indicated in the figures for each experiment.

Figure S6 | Full gel image of Figure 3B.

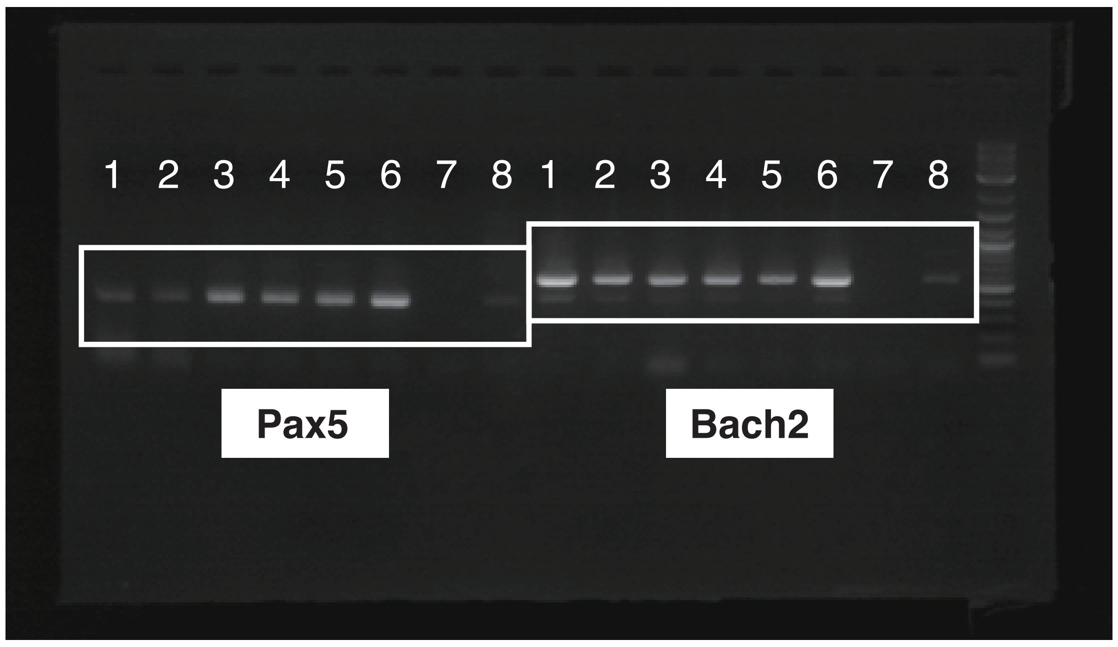

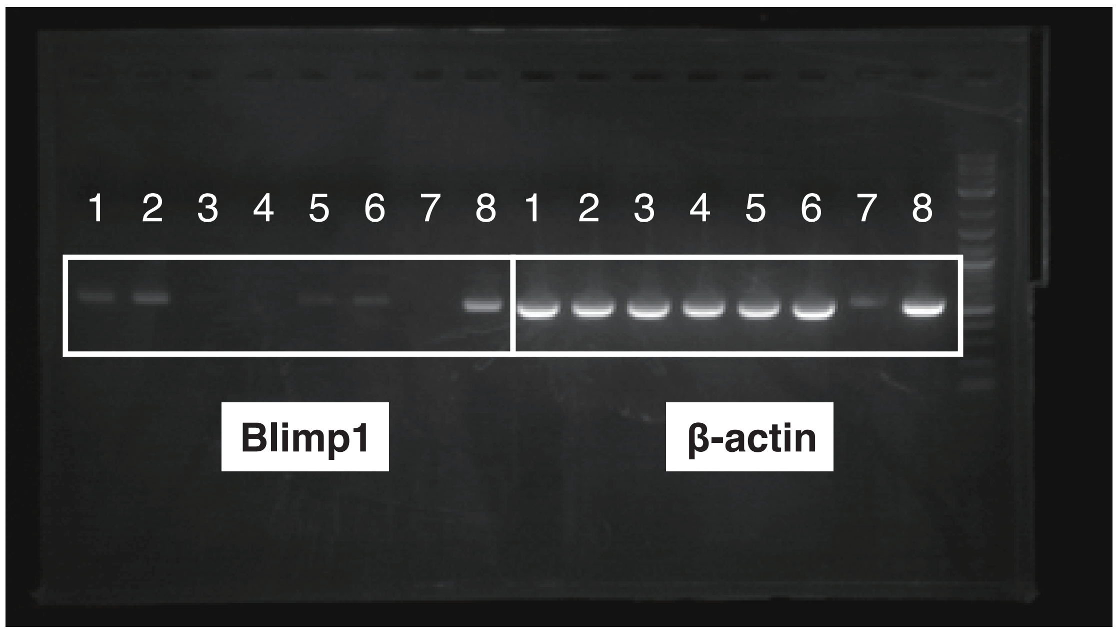

1: IgM+ B cell, 2: IgG1+ B cell, 3: IgM+ CB, 4: IgG1+ CB, 5: IgM+ CC, 6: IgG1+ CC, 7: Water, 8: ASC
